# Natural Mineral Waters as Solvents for Sustainable Extraction of Polyphenolic Compounds from Aronia Stems

**DOI:** 10.3390/foods15020406

**Published:** 2026-01-22

**Authors:** Irina-Loredana Ifrim, Ionuț Avătămăniței, Oana-Irina Patriciu, Cristina-Gabriela Grigoraș, Adriana-Luminița Fînaru

**Affiliations:** Department of Chemical and Food Engineering, “Vasile Alecsandri” University of Bacau, 157 Marasesti Street, 600115 Bacau, Romania; avatamanitei.ionut@ing.ub.ro (I.A.); cristina.grigoras@ub.ro (C.-G.G.); adrianaf@ub.ro (A.-L.F.)

**Keywords:** natural mineral water, aronia, by-products, eco-friendly extraction, phytochemicals, sustainability

## Abstract

*Aronia melanocarpa,* a plant with nutrient-rich fruits, with application in the food and pharmaceutical industry, has been extensively investigated but, nevertheless, the exploration of the secondary metabolites profile from its by-products remains quite limited. The main objective of this study was to evaluate the possibility of using some different natural mineral waters from Romania, as green solvents, for the extraction of bioactive compounds from aronia stems and fruits by applying eco-compatible working techniques (maceration for 24 h, and ultrasonication at room temperature and 50 °C for 30 min). The effect of five natural mineral waters (one with medium and four with low mineral content) on the extraction capacity and phytochemical profile of stems and fruits’ extracts was monitored using fast and efficient analysis techniques (electrochemical, spectroscopic, and chromatographic) and compared with that of classical solvents. The results showed that, in the case of stems, extraction by maceration was, for all types of water used, the most efficient, followed by ultrasonication at room temperature. Also, at the same time, in most cases, all mineral waters showed better performance than distilled water, and the highest efficiency of the extraction process was recorded for natural water with a medium mineralization level. The similarity observed in the phytochemical profiles of aqueous extracts from the aronia stems and the fruits highlights both the potential of this by-product as a source of bioactive compounds and the efficiency of natural mineral waters as green extraction solvents.

## 1. Introduction

Plants are considered rich sources of biologically active compounds with antioxidant, antibacterial, antifungal, anti-inflammatory, and antitumor effects, etc. On the other hand, plant by-products, usually used for low-value applications, can also constitute a relevant source for various bioactive compounds (primary and secondary metabolites), useful in obtaining cosmetics, pharmaceuticals, supplements, or food products valuable in improving human nutrition, health, and well-being [1].

One of the most known and studied classes of bioactive compounds is represented by the polyphenols. These secondary metabolites have proven various nutritional values and beneficial effects on health due to their antioxidant and anti-inflammatory properties [2,3,4,5].

Black chokeberry (*Aronia melanocarpa*), also called aronia, a species of shrubs belonging to the *Rosaceae* family, native to eastern parts of North America, is cultivated around the world for its medicinal and nutritional value. Aronia, considered a superfruit, has a positive impact on health due to its rich and varied content of bioactive components, such as vitamins, minerals, and polyphenolic compounds.

Its anti-inflammatory, antibacterial, hypolipidemic, hypoglycemic, anticancer, antidepressant, and antifatigue effects promoted it over the past decade as a raw material for a wide variety of juices, wines, teas, effervescent tablets, and nutritional supplements [6,7,8,9,10,11,12].

While chokeberry fruits are well characterized from a compositional point of view, their by-products (leaves, stems, etc.) have been less studied, with the number of investigations on this subject being limited [13,14,15].

Nowadays, there is growing interest in obtaining bioactive compounds from plants, residues of food industries, and by-products through green methods [16,17,18,19]. In this regard, one of the biggest challenges for the recovery of secondary metabolites from plants is to develop an appropriate working system (equipment, extraction protocol, and solvent), simultaneously compatible with the chemical complexity of plant by-products and ecologically sustainable.

The efficiency of the extraction process, conducted through conventional (decoction, infusion, maceration, Soxhlet extraction, percolation, etc.) or modern methods (ultrasonication, microwave-assisted extraction, extraction with pressurized liquids, extraction with subcritical or supercritical fluids and accelerated solvent extraction, electrotechnologies such as pulsed electric field and high-voltage electrical discharges, enzyme-assisted extraction, etc.), is influenced by numerous factors related to the solvent, the composition of the plant material, and to the extraction process parameters (time, temperature, plant–solvent ratio, etc.) [1,20,21,22].

Taking into account the concept of green chemistry, lately, attention has been directed toward the use of non-toxic solvents that do not harm human health and the environment. Among them, water can be considered the most risk-free and the least expensive solvent.

Only few studies regarding the efficiency of extraction techniques for biologically active compounds using natural mineral waters and the influence of their composition on the biological activity of the obtained extracts are available [23,24,25].

On the other hand, a series of previous studies indicate that factors such as pH value or presence of dissolved salts (especially those of calcium and magnesium) and bicarbonate ions can affect the bioactive compounds’ extraction, particularly that of polyphenols [24,26].

In Romania, there are over 2000 mineral water springs (over 40% of the European reserve of natural mineral waters [27]), whose chemical diversity is given by the particularly complex geological conditions in which they were formed. Important Romanian mineral water deposits are located in mountainous areas and in intermountain depressions, far from the pollution sources characteristic of industrial areas or where intensive agriculture is practiced. Most springs presenting carbonated mineral waters are bottled and commercially available. When consumed with food or shortly before a meal, they stimulate digestive functions and excite gastric secretion and motility, intestinal, pancreatic, and biliary secretions, favoring the acceleration of food absorption.

The beneficial role of natural mineral waters on the healthy constitution and functioning of the human body is amplified by their content in mineral salts, and by their purity, characterized by the total absence of artificial contaminants. These waters are rich in minerals, such as calcium (Ca), magnesium (Mg), sodium (Na), and potassium (K), and ions, such as bicarbonate (HCO_3_^−^), chlorine (Cl^−^), and sulfate (SO_4_^2−^). At the trace level, metals and metalloids, such as manganese (Mn), iron (Fe), silicon (Si), selenium (Se), and zinc (Zn), are also present.

Our research concerns are mainly focused on the superior valorization of regional resources (NE Romania), namely, the natural mineral waters in the area [28,29,30], as well as the diversity of plant-based foods and their by-products as potential sources of bioactive compounds [31,32,33,34].

From this perspective, the present work was designed as a qualitative and comparative study concerning the potential of commercial natural mineral waters of Romania to efficiently extract polyphenols, especially from aronia by-products (stems) but also from fruits, in order to initiate future research on their functionalities and bioactivities. The effects of the environmentally friendly solvents used and of the extraction techniques (maceration and ultrasound) were evaluated by safe, efficient, and less expensive analytical methods (electrochemical, spectroscopic, and chromatographic methods). The use of instrumental fingerprinting techniques (UV-Vis and FTIR spectroscopy) was considered for a rapid screening of the extracts’ phytochemical profiles in order to highlight differences induced by the solvent type and the extraction method.

## 2. Materials and Methods

### 2.1. Plant Material

**Figure 1:**
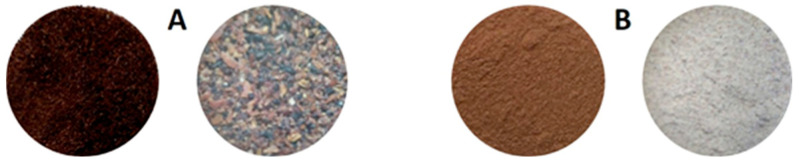
Macroscopic and stereomicroscopic images of powdered aronia ((**A**) fruits and (**B**) stems).

Aronia fruits and stems used in the experiments were collected in the hilly area of Bacau County, northeast region (latitude: 46.598553 N; longitude: 26.843002 E) of Romania, in August 2024. They were naturally dried in a cool and dark place until reaching a moisture content of 10–12%. Then, the fruits (F) were separated from stems (S), packed in sealed glass containers, protected with aluminum foil, and stored in a dark and cool place. Before use, they were grinded with an electric grinder (Heinner, model HCG-150SS, 150 W, Heinner, Bucharest, Romania). The resulted powders were sieved through a digital electromagnetic sieve shaker (FILTRA Vibratión FTS-0200, Barcelona, Spain). Fractions with size of 0.200 mm for stems and 0.250 mm for fruits were retained for extraction. Figure 1 shows the macroscopic photographs together with images obtained under stereomicroscope (Optika Microscopes, Model ST-30FX, Ponteranica, Italy) of the vegetal material in powder form.

### 2.2. Extraction Solvents

- Ethanol 96° (Chemical Company S.A., Iași, Romania) was mixed with distilled water (40/60 *v*/*v*) and used for hydroalcoholic extraction (HA).
- Distilled water (*aqua destillata*, AD) and five commercial natural mineral waters (NMWs) were used for aqueous extraction.
- The sources of natural mineral waters, one with medium mineral content (500–1500 mg/L) and four with low mineral content (50–500 mg/L) [35], were as follows:
  1. Tușnad (T), source FH 35bis, Tușnad, Harghita, carbonated.
  2. Aquavia (AQV), source A1—A2R, Bologa, Cluj, non-carbonated.
  3. Aqua Carpatica (AC), source AQUA, Broșteni, Suceava, non-carbonated.
  4. Aqua Carpatica Kids (ACK), source Izvor BAJENARU, Paltiniș, Suceava, non-carbonated.
  5. Perla Harghitei (PH), source FH Artezia 2, Sânsimion, Harghita, non-carbonated.

The main characteristics included on the labels of commercial mineral waters used in extraction are presented in Table 1 (according to the producers’ statements).

### 2.3. Extraction Procedure

Two grams of powdered plant sample and 40 mL of solvent were submitted to 30 min extractions performed by two techniques: maceration (M) at room temperature for 24 h and ultrasonication (US) in a digital ultrasonic cleaner, Biobase model UC-40A (Biobase Biodustry (Shandong) Co., Ltd., Jinan, China), at room temperature (USRT) and at 50 °C (US50). The extracts were separated by filtration through Whatman filter paper No. 42 (Marlborough, MA, USA). The resulted 42 extracts (Table 2) were stored at 4 °C until further analyses.

### 2.4. Solvent and Extract Characterization

The electrochemical parameters, pH, conductivity (EC), salinity (SAL), and total dissolved solids (TDSs), of solvents and extracts were measured using a Thermo Scientific™ Orion™ Versa Star Pro™ Multiparameter Benchtop Meter (Thermo Fisher Scientific, Waltham, MA, USA) equipped with a ROSS Ultra pH/ATC electrode and DuraProbe conductivity cell 013005MD.

The extracts were further analyzed through ultraviolet-visible (UV-Vis) and Fourier transform infrared (FTIR) spectroscopy. In the first case, a UV-Vis spectrophotometer (Shimadzu UV-1280, Kyoto, Japan) was used for scanning the samples in the wavelength ranging from 190 to 1100 nm. In the second case, the analyses were performed with an IRSpirit FTIR spectrometer with attenuated total reflectance single-reflection (QATR-S) auxiliary (Shimadzu, Bucharest, Romania) employed between 4000 and 400 cm^−1^ (50 scans/min) with a resolution of 8 cm^−1^. The cleaning of the QATR-S accessory was realized with ethanol. The high-performance thin-layer chromatography (HPTLC) technique was used for a rapid screening of the extracted compounds [36,37] using a CAMAG HPTLC system (CAMAG, Muttenz, Switzerland) equipped with a Semi Auto Sample Applicator CAMAG^®^Linomat 5, TLC Visualizer, CAMAG TLC Scanner 3 UV with visionCats CAMAG HPTLC software (version 3.0). The HPTLC plates (20 cm × 10 cm silica gel 60 F254—Merck, Darmstadt, Germany) were used without any pretreatment. The length of the bands was 8 mm, the application rate was 5 µL/s, and the application volume was 2 µL. The plates were developed with an eluent adapted from Asfaw et al. [38] containing toluene:ethyl acetate:ethanol:formic acid (20:12:8:4 *v*/*v*/*v*/*v*). After development, the plates were removed, dried at room temperature, and visualized under UV light at 254 nm and 366 nm.

### 2.5. Statistical Analysis

All the experiments were performed in triplicate, and the obtained results were reported as mean values with standard deviation (mean ± SD). The variables’ relationship was tested using linear regression and quantified by calculating the Pearson correlation coefficient. Microsoft Office Excel 2016 (Microsoft, Redmond, WA, USA) was used for this purpose. Principal component analysis (PCA), permutational multivariate analysis of variance (PERMANOVA), and partial least squares–discriminant analysis (PLS-DA) were carried out with MetaboAnalyst 6.0.

## 3. Results and Discussion

The aqueous and hydroalcoholic extracts were obtained through two extraction techniques, maceration and ultrasonication.

Maceration, although a traditional, simple, and inexpensive method that allows the solvent to gradually diffuse into plant tissues, extracting bioactive compounds through a concentration gradient, requires a long interaction time [21,22].

Ultrasonication is an accessible, modern, rapid, and non-polluting extraction technique that helps to release bioactive compounds from plant materials to a solvent, exposed to high-frequency sound waves. The process relies on ultrasonic waves to create cavitation bubbles in the liquid. As these bubbles collapse, they generate intense localized heat and pressure that can be harnessed to facilitate the extraction process [21,39,40].

Aqueous and hydroalcoholic extracts of aronia fruits and stems were subjected to analyses through physicochemical, UV-Vis, FTIR, and HPTLC evaluations.

### 3.1. Electrochemical Analyses

**Figure 2:**
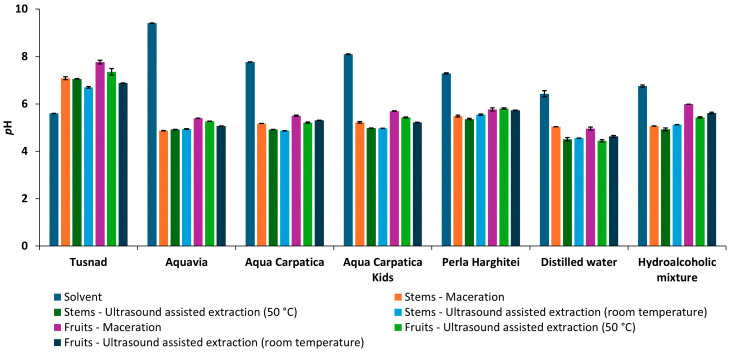
pH of solvents and extracts obtained from aronia (stems and fruits).

**Figure 3:**
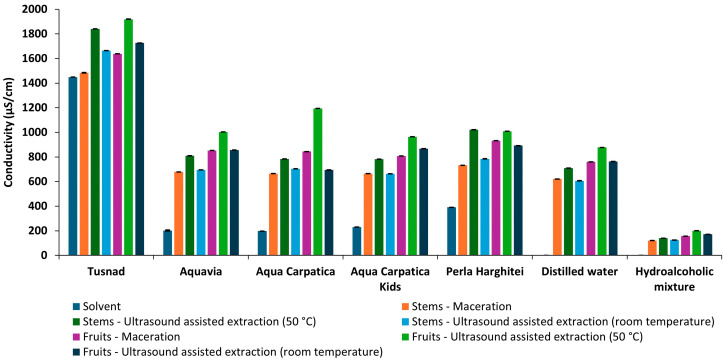
Conductivity of solvents and extracts obtained from aronia (stems and fruits).

**Figure 4:**
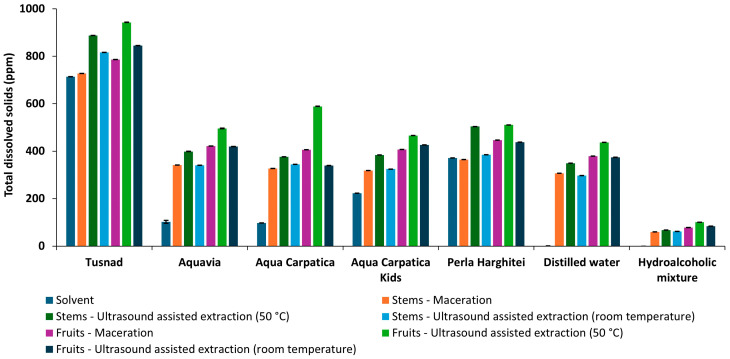
Total dissolved solids of solvents and extracts obtained from aronia (stems and fruits).

**Figure 5:**
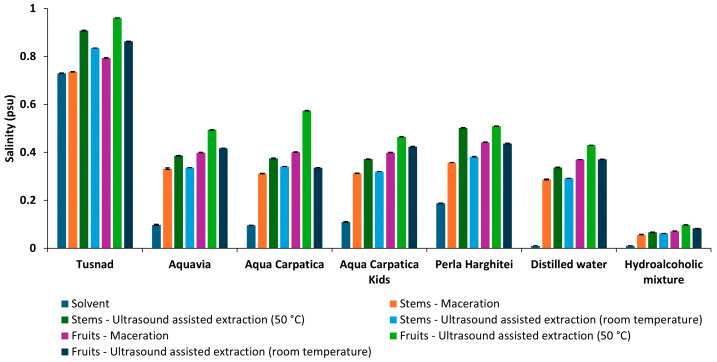
Salinity of solvents and extracts obtained from aronia (stems and fruits).

Both the solvents and the crude extracts of aronia fruits and stems were analyzed by physicochemical methods. The results obtained in terms of representative physicochemical parameters, such as pH, electrical conductivity (EC), total dissolved solids (TDSs), and salinity (SAL), are summarized in Figure 2, Figure 3, Figure 4 and Figure 5. Data are shown as mean ±SD.

#### 3.1.1. pH

The pH of the natural mineral waters used in the experiment ranged from 5.60 ± 0.002 (T) to 9.41 ± 0.002 (AQV).

Regarding the pH values, extracts from both the stems and the fruits obtained using non-carbonated mineral waters (AQV, AC, ACK, and PH), distilled water, and hydroethanolic mixture were acidic, while the extracts obtained using carbonated mineral water (T) were neutral.

As shown in Figure 2, a decrease in the pH of the extracts compared to the initial solvents was observed (both in the case of non-carbonated natural waters with low mineral content, AQV, AC, ACK, and PH, from the basic to the acidic range, and in the case of AD and HA), with the exception of Tușnad (carbonated natural water with medium mineral content), where an opposite behavior was seen.

The smallest pH variations were recorded in stem extracts when using Tușnad natural mineral water as a solvent (1.1−1.5 pH units) and in hydroalcoholic fruit extracts (0.8−1.2 pH units). Also, in the case of AQV natural mineral water, the pH variation was the highest, registering a decrease between 4 and 4.5 pH units for both stem and fruit extracts, both macerated and ultrasonicated.

In terms of pH, the values for stem extracts were slightly lower than the ones for fruits.

#### 3.1.2. Conductivity

Among all the extracts obtained (Figure 3), the lowest conductivity values were presented by the hydroalcoholic ones (in the range of 121.10 ± 0.264 to 200.71 ± 0.065 µS/cm), followed by those in distilled water, then relatively close values were presented by the extracts in ACK, AC, and AQV, followed by the PH extracts, while the extracts with T had significantly higher conductivity values, between 1484.33 ± 2.52 and 1919.33 ± 2.08 µS/cm.

Considering the low ion content of distilled water and hydroethanolic solution, the largest increases in conductivity were observed in the case of extraction with the two solvents (about 550-fold and 240-fold higher, respectively), which denotes a much better extraction power of ions from plant material compared to natural mineral waters.

In the case of mineral waters as solvents, conductivity increases were much smaller, ranging between 1.15 and 4.6 times. The smallest increase was recorded for Tușnad natural mineral water, the only carbonated water, classified as water with a medium mineral content, while for the most alkaline mineral water, AQV, the variation was the largest.

For all samples, the ultrasonic extraction method at 50 °C favored an increase in the rate of ion solubilization, their mobility, and, therefore, their concentration in solution, inducing a greater increase in conductivity compared to maceration and ultrasonication at room temperature.

Due to the higher mineral content of fruits compared to stems [13,41], it was expected that the increases in the conductivity values of the extracts would also be greater for fruits.

#### 3.1.3. Total Dissolved Solids (TDSs) and Salinity (SAL)

In accordance with conductivity, the trends of TDS and salinity measurements were similar to those of conductivity (Figure 4 and Figure 5).

The Pearson correlation coefficients (Table 3) were calculated for physicochemical parameters of the extracts obtained using the five natural mineral waters (T, AQV, AC, ACK, and PH), distilled water (AD), and hydroalcoholic mixture (HA).

Very strong correlations between EC and TDS (over 0.99) and EC and SAL (over 0.95) can be observed for all extracts. The highest negative value of coefficient correlation, pH–EC (−0.8869), pH–TDS (−0.9014), and pH–SAL (−0.888), can be observed for the extracts with PH natural mineral water. In the case of the most alkaline mineral water (AQV), weak positive correlations of pH with the other parameters were recorded (0.229–0.347).

### 3.2. Phytochemical Profiles

#### 3.2.1. FTIR Analysis

**Figure 6:**
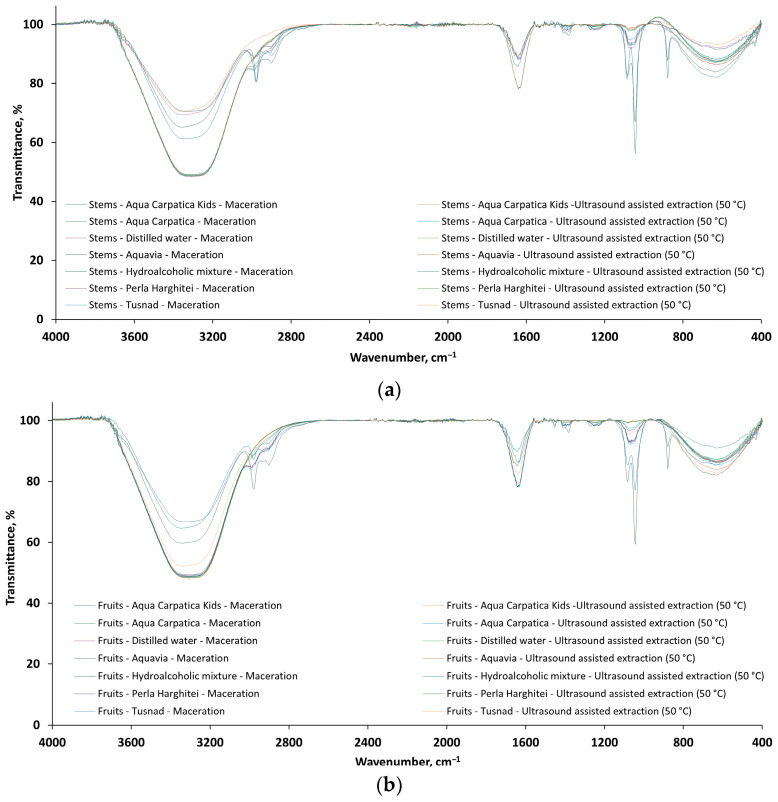
The FTIR spectra of the 28 extracts from aronia: (**a**) S—stems and (**b**) F—fruits.

The FTIR spectra for 28 extract samples of *Aronia melanocarpa*, 14 stem extracts (Figure 6a: 7/S-M and 7/S-US50) and 14 fruit extracts (Figure 6b: 7/F-M and 7/F-US50), respectively, recorded in the wavenumber range 4000–400 cm^−1^, are presented comparatively in Figure 6.

From Figure 6, it can be seen that the shape of the FTIR spectra for both stem (Figure 6a) and fruit (Figure 6b) extracts is similar and, in accordance with previous studies [42,43,44,45,46,47], shows absorption bands for functional groups at frequencies characteristic of polyphenolic components usually present in aronia. The dominant bands present in the FTIR spectra of the 28 analyzed extracts are given in Table 4.

Additionally, the weak and small peaks observed at 2990/2954 (stem extracts) and 2980/2897 (fruit extracts) where possibly originating from –CH, –CH_2_, and –CH_3_ groups (stretching vibrations of C–H bonds from carbohydrates). The peaks around 1400 cm^−1^ and 1250 cm^−1^ can be assigned to phenolic C–O stretching of the pyran nucleus, typical for flavonoid C-rings [42,45].

#### 3.2.2. UV-Vis Analysis

**Figure 7:**
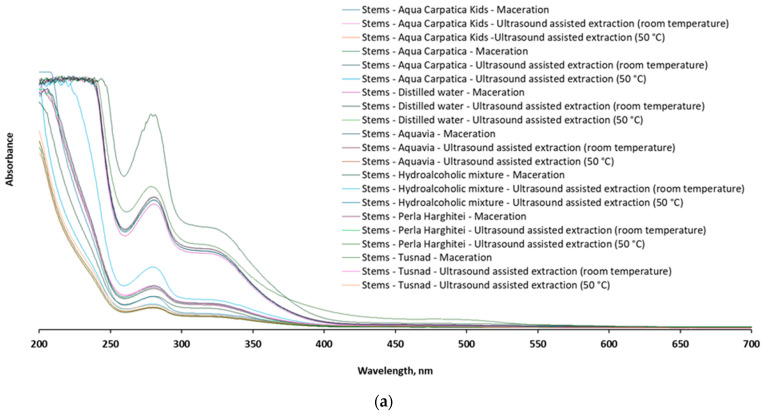

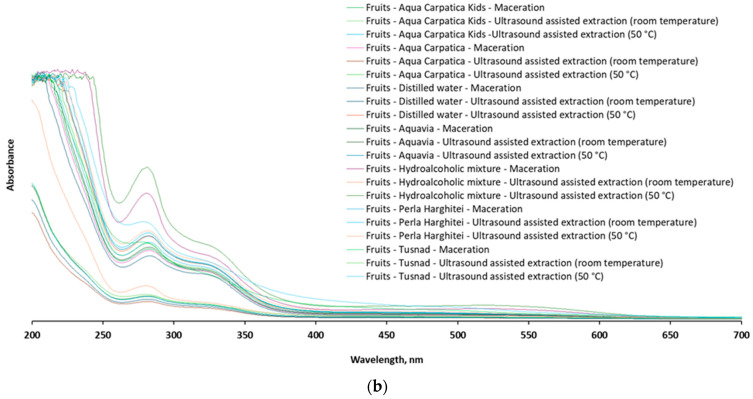
The UV-Vis profile of the 42 extracts from aronia: (**a**) stems and (**b**) fruits.

After UV-Vis scanning of the samples, a comparative spectrophotometric fingerprint of the chokeberry fruit and stem extracts was recorded in the representative range 200–700 nm (Figure 7).

The qualitative profiles of the UV-Vis spectra recorded for both stem and fruit extracts, respectively, the position (λ_max_) and intensity of the spectral bands, are similar and in full agreement with other available data reported for the content of polyphenolic compounds in chokeberry [8,14,15,48,49,50,51,52,53], confirming at the same time the fact that stem extracts also constitute a valuable source of such bioactive compounds.

From the examination of the spectral profile, it is observed that the extracts from the stems are also characterized by the presence of intense absorption bands in the ranges of 270–290 nm and 320–340 nm, respectively, considered specific to different types of phenolic acids and flavonoids, while the signals in the range of 480–520 nm, attributed mainly to anthocyanins [49,50], are absent in the case of the stems or much less represented (C-T-M: 470–780 nm) compared to those in the fruit spectrum.

**Figure 8:**
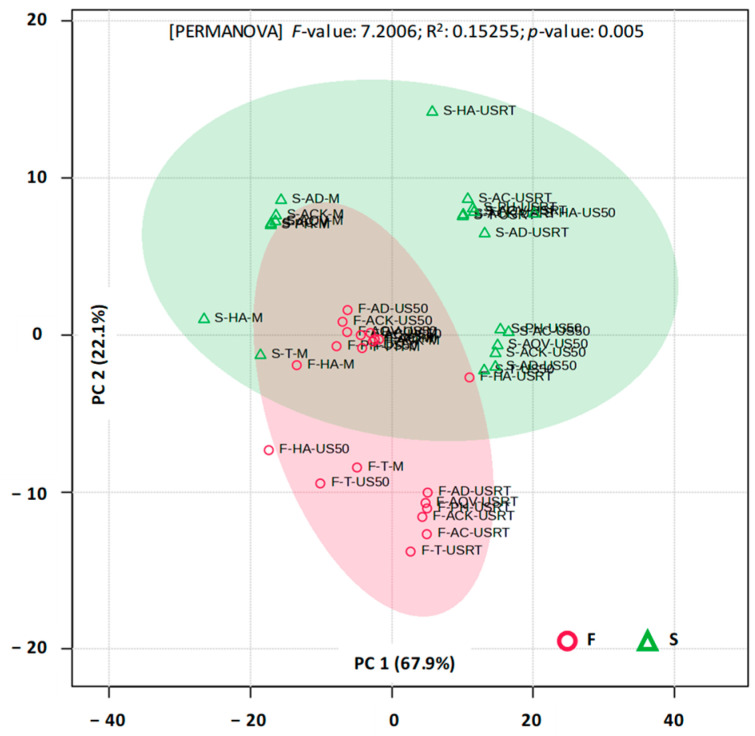
2D PCA score plot for UV-Vis (200–700 nm) profiles of aronia stems (S) and fruits (F). ACK—Aqua Carpatica Kids; AC—Aqua Carpatica; AD—aqua destillata (distilled water); AQV—Aquavia; HA—hydroalcoholic mixture; PH—Perla Harghitei; T—Tusnad; M—maceration; USRT—ultrasound-assisted extraction (room temperature); US50—ultrasound-assisted extraction (50 °C).

PCA (Figure 8) performed on the full UV-Vis spectra (200–700 nm) differentiates aronia fruit extracts from those obtained from stems.

The first two principal components explained 90% of the total variance. The separation was statistically confirmed by PERMANOVA, demonstrating that the plant material significantly influences the overall spectral profile. PLSDA further supported this observation (the first two components jointly explained 89% of the variance), indicating that multiple spectral regions contribute to fruit–stem discrimination rather than a single dominant absorbance feature. Based on these results, it can be inferred that the two plant matrices should not be treated interchangeably when evaluating the extraction of bioactive compounds. In order to confirm or invalidate this hypothesis, a separate multivariate analysis was conducted. PCA and PLSDA realized for UV-Vis spectra of fruit and stem extracts revealed no significant differences for wavelengths between 200 and 700 nm. On the other hand, various differences were detected when the analyses were restricted to the regions specific for phenolic acids (270–290 nm), flavonoids (320–340 nm), and anthocyanins (480–520 nm).

**Figure 9:**
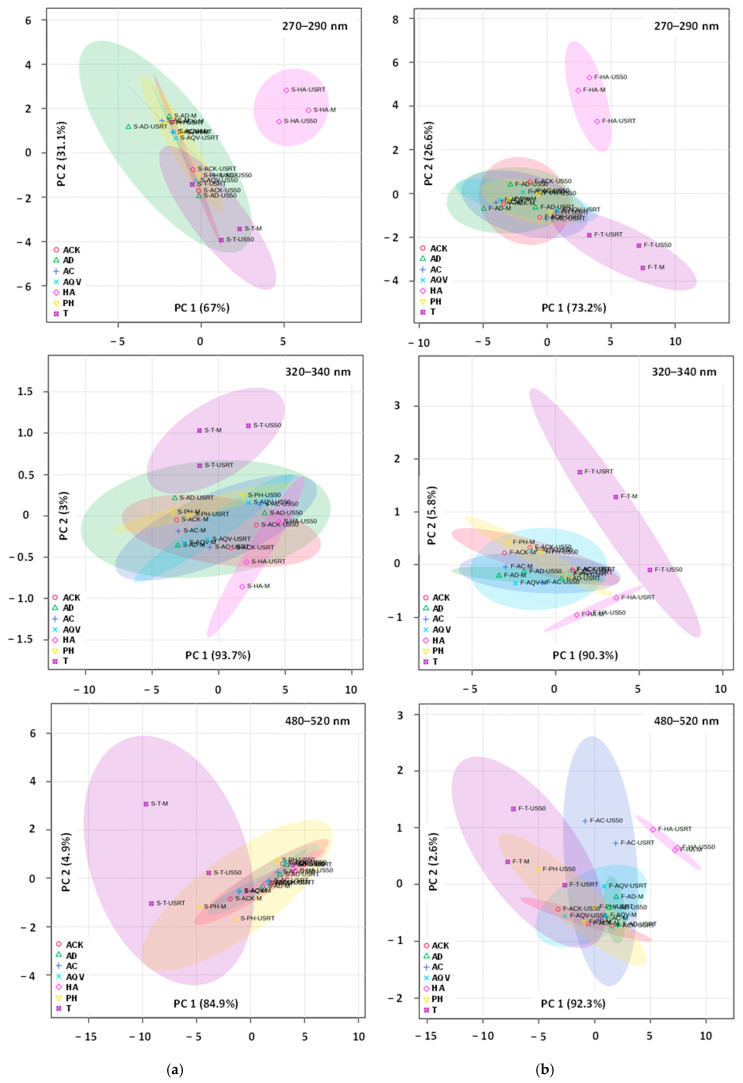
2D PCA score plot for UV-Vis profiles of aronia stems (**a**) and fruits (**b**). ACK—Aqua Carpatica Kids; AC—Aqua Carpatica; AD—aqua destillata (distilled water); AQV—Aquavia; HA—hydroalcoholic mixture; PH—Perla Harghitei; T—Tusnad; M—maceration; USRT—ultrasound-assisted extraction (room temperature); US50—ultrasound-assisted extraction (50 °C).

For aronia stems (Figure 9a), PCA and PERMANOVA highlighted differences in multivariate structures among the individual sample types (ACK, AD, AC, AQV, HA, PH, and T) and across analytical configurations. In the first analysis (270–290 nm), PCA revealed strong separation among sample groups, with the first two components explaining 98.1% of the total variance. HA samples formed a distinct cluster, separated from the remaining sample types, while ACK, AD, AC, AQV, PH, and T were differentiated along both PC1 and PC2, with T samples showing greater internal dispersion. This structuring was sustained by PERMANOVA results (F-value = 7.21, R^2^ = 0.7555, *p*-value = 0.002). The second PCA (320–340 nm) showed that although PC1 explained 93.7% of the variance, AC, AQV, ACK, AD, PH, and HA samples largely overlapped, and T samples were not consistently separated from the other groups, reflecting weak discrimination among sample types. The lack of structure was consistent with the PERMANOVA data (F-value = 0.8936, R^2^ = 0.2769, *p*-value = 0.537). In the third analysis (480–520 nm), multivariate separation was again evident (PC1 84.9% and PC2 4.9%). T samples shifted toward negative PC1 values and exhibited extensive dispersion.

The three PCA score plots for aronia fruits (Figure 9b) revealed a consistent pattern of multivariate differentiation among groups, with separation mainly driven along PC1 (73.2%, 90.3%, and 92.3%, respectively) and a more reduced contribution recorded for PC2. In the first and third plots, several groups formed separated and compact clusters. The overlap was limited with HA and T ellipses clearly displaced along PC1 and/or PC2. ACK, AD, AC, AQV, and PH showed varying degrees of overlap, suggesting partial similarity and shared variance, though their centroids remained distinguishable, especially when PC2 was considered. The PCA plot specific to the 320–340 nm UV-Vis range showed a greater overlap among most groups and a visibly broader dispersion of ellipses, indicating weaker yet noticeable structuring. These remarks are in agreement with PERMANOVA results, which were statistically significant (F-value = 11.9, 3.76, and 10.16; *p*-value = 0.001, 0.019, and 0.001) for all three wavelength ranges, confirming that group centroids differ beyond random expectation. The R^2^ values (0.8324, 0.6171, and 0.8132) indicate that group identity explains a substantial proportion of total multivariate variance, particularly in the cases of 270–290 nm and 480–520 nm. PLSDA, with the first two components jointly explaining 99.8%, 95.6%, and, respectively, 94.6% of the variance, validated these outcomes.

AC, AQV, ACK, AD, PH, and HA samples clustered and followed a structured gradient, with only partial separation. This pattern was statistically supported by PERMANOVA (F-value = 5.65, R^2^ = 0.7078, p-value = 0.003), confirming that differences among sample types, particularly the distinct behavior of T and HA relative to AC, AQV, and AD, were dominant in the multivariate variation. PLSDA sustained these observations, with the first two components jointly explaining 98.1%, 96.6%, and, respectively, 88.3% of the variance.

The multivariate statistical analyses applied to the UV-Vis spectra of aronia stem and fruit extracts emphasize that the extraction technique, particularly when combined with appropriate solvents, enhances the recovery of bioactive compounds. The discrimination among mineral waters suggests that ionic composition and mineral content influence the extraction performance, particularly for phenolic acids. This effect is more prominent in targeted spectral regions than for the full UV-Vis range, reinforcing the importance of chemically selective analysis.

#### 3.2.3. HPTLC Analysis

In addition to the spectrophotometric analysis, HPTLC testing of the aronia fruit and stem extracts was also performed. HPTLC is a relatively simple, inexpensive, and reproducible testing method that can provide essential information on the comparative compositional quality of plant extracts.

HPTLC silica gel plates were developed with toluene:ethyl acetate:ethanol:formic acid (20:12:8:4 *v*/*v*/*v*/*v*), and then the chromatograms were scanned at 254 nm and 366 nm.

The captured HPTLC plate images were converted into densitograms.

**Figure 10:**
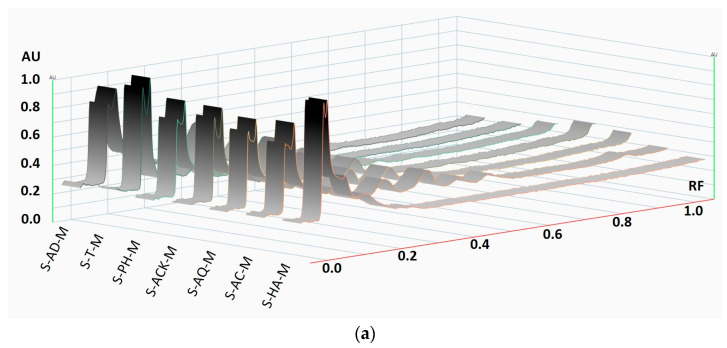

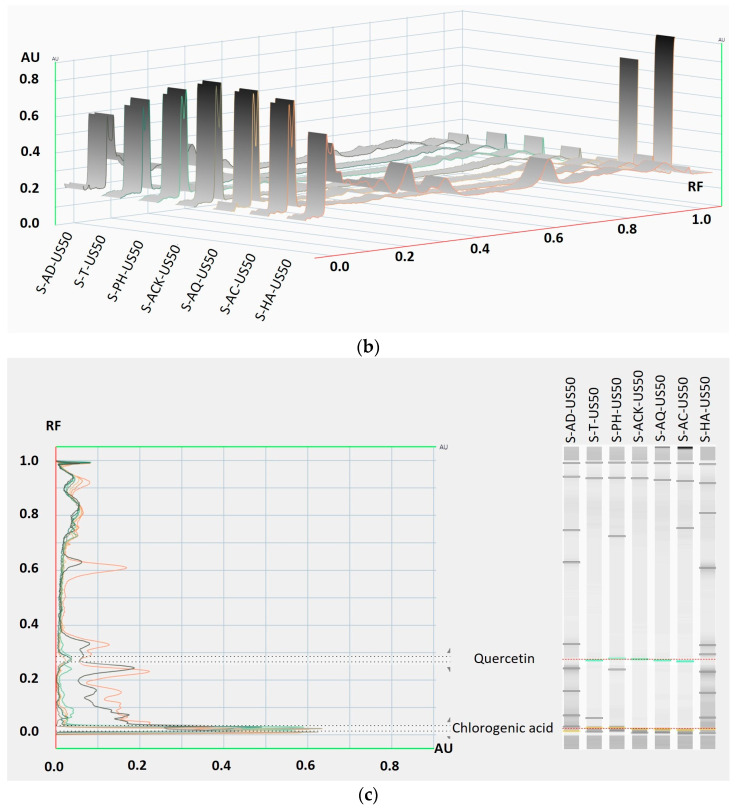
HPTLC profile of aronia stem extracts at 366 nm: densitograms of the stem extracts obtained by (**a**) maceration, (**b**) ultrasonication at 50 °C, and (**c**) the tracks of 7 extracts’ US50 bands attributed to quercetin and chlorogenic acid, compared to reference compound mixtures.

From the qualitative profiles of the aronia stem extracts (Figure 10), it can be seen that the extraction fingerprint using natural mineral waters as solvents is similar to the case of using classic solvents, such as distilled water or hydroalcoholic mixture.

According to other studies [54], it must be noted that both the extraction conditions and the nature of the solvent influence the profile of aronia extracts, and among the representative polyphenolic compounds, the assignment of bands was performed using the standards for quercetin and chlorogenic acid [13,14,55,56].

### 3.3. The Influence of Solvents and Experimental Methodology on the Extraction Process of Bioactive Compounds

The results obtained from spectrophotometric and chromatographic analyses highlight the fact that regardless of the working technique used (M or US), the efficiency of the extraction process in the presence of natural mineral waters is superior to distilled water in most cases for both stems and fruits.

FTIR and UV-Vis analyses were employed to qualitatively compare spectral profiles and identify characteristic absorption bands, rather than to perform quantitative spectral analysis. The obtained spectra showed a high degree of overlap, with only negligible variations in band position and relative intensity. This fact visually demonstrates the reproducibility of the extraction and analytical procedures and the qualitative similarity among replicates.

The best performance recorded for all extracts using the hydroalcoholic mixture (water/ethanol: 40/60 *v*/*v*) confirms the increased capacity to solubilize polyphenolic compounds in such environments. However, the use of ultrasound brings the extraction capacity of some mineral waters (ACK, AC, and T) closer to that of HA.

Thus, in the case of stems, maceration proved to be more efficient, followed by USRT for all natural mineral waters, while under US50 conditions only two out of the five mineral waters (AC and T) showed an extraction capacity superior to distilled water, with the other three (ACK, AQV, and PH) having behavior closer to that of AD.

In contrast, for fruits, the efficiency of the extraction process under the action of ultrasound and temperature (US50) is noteworthy, followed by M and USTA. In all working conditions, of the five mineral waters, Tușnad water proved to be the most efficient solvent.

Regarding the behavior of different waters used in the extraction of bioactive compounds, previous studies have reported that the presence of alkaline and alkaline earth metal cations in small quantities, respectively, low-mineralized waters, caused an increase in extraction efficiency [24,25]. Physicochemical parameters, such as pH, electrical conductivity (EC), total dissolved solids (TDSs), and salinity, jointly reflect the ionic strength and buffering capacity of the solvent, which can influence solvent–solute interactions, cell wall permeability, and the stability of the extracted compounds. Polyphenols are known to be sensitive to pH variations, with an acidic environment being favorable and known as limiting the oxidative degradation.

The pH also influences, both qualitatively and quantitatively, the extraction process of polyphenolic compounds, with a low pH value stabilizing the concentration of polyphenols [26,57].

Although the initial pH values of all the solvents used ranged from acidic to alkaline (T: 5.60, AD: 6.43, HA: 6.76, PH: 7.29, AC: 7.77, ACK: 8.10, and AQV: 9.41), the pH of the resulting extracts converged toward acidic (AQV, AC, ACK, and PH), and distilled water and hydroethanolic mixture were acidic (4.44–5.99) or neutral values (6.70–7.77 for the carbonated mineral water—T), which may have contributed to the preservation of phenolic structures during extraction.

According to these results, all non-carbonated natural waters with low mineral content presented a positive influence on the extraction process (ACK ≥ AC > AQV ≥ PH) compared with distillated water regardless of the methodology of extraction and plant material used. Even if the initial pH range of these waters was 7.29–9.41, the pH values of the obtained extracts varied between 4.87 and 5.81.

The increase of EC, TDSs, and SAL after extraction is an indicator for the solubilization of ionic species and plant-derived compounds [23,24]. Distilled water and the hydroalcoholic mixture exhibited the largest relative increases in conductivity due to their initially low ionic content, while for natural mineral waters moderate variations were observed, suggesting a buffering effect associated with their intrinsic mineral composition.

The Pearson correlation coefficients calculated for the physicochemical parameters (pH, EC, TDSs, and SAL) helped us to create an image regarding the relationship between these indicators during the extraction process. The efficiency of the electrochemical method, for monitoring such closely related parameters as EC, the amount of TDSs, and salinity, for which the Pearson correlation coefficient value was close to unit for all extracts obtained, respectively, EC–TDS (over 0.99) and EC–SAL (over 0.95), should be noted.

Differences detected among the natural mineral waters can be linked to the mineralization level and ionic composition. Thus, the presence of ions, such as calcium, magnesium, and bicarbonate, may enhance the extraction of phenolic compounds by modifying ionic strength, facilitating mass transfer, or stabilizing phenolic structures through weak complexation mechanisms [24,25,52]. The comparatively better extraction behavior observed for Tușnad water, characterized by medium mineralization and dissolved CO_2_, may result from a combined effect of mineral content, buffering capacity, and pH modulation.

It should be emphasized that since the present study provides qualitative and comparative evidence, a quantitative assessment of the contribution of individual ions remains to be carried out to confirm the above hypothesis.

## 4. Conclusions

In the context of sustainable development, the use of plant by-products through the superior valorization of their bioactive compounds and green extraction methods is essential.

The extraction of biologically active compounds from aronia by-products was evaluated in the present study in terms of its dependence on the level of mineralization of the natural mineral waters used as solvents, and the Pearson correlation coefficients were calculated for physicochemical parameters (pH, EC, TDSs, and SAL).

The results obtained from UV-Vis, FTIR, and HPTLC analyses revealed that the similar phytochemical profiles of both the stem and fruit extracts indicates a good extraction capacity in the case of natural mineral waters, most of the time superior to distilled water, regardless of the extraction conditions (maceration and ultrasonication at room temperature). For ultrasonication at 50 °C, only two out of the five mineral waters (AC and T) showed an extraction capacity superior to distilled water.

Moreover, this study highlighted the potential of combining spectroscopic analysis techniques (UV-Vis and FTIR) with chromatographic (HPTLC) and electrochemical ones to obtain the phytochemical fingerprint of the extracts. The existence of a significant content of active principles in the stem extracts was also reconfirmed.

The efficiency of the ultrasonic extraction technique was likewise observed, with the extraction process taking place with very good results, in a short time (30 min), at RT for stems and at 50 °C for fruits, compared to those obtained after 24 h by maceration.

It was also noted that Tușnad water, carbonated and with a medium mineral content, was the most efficient extraction solvent among all the waters analyzed.

The absence of organic solvent residues, combined with the food-grade nature of the solvent, allows the resulting extracts to be potentially used directly in food, nutraceutical, or cosmetic formulations. The approach proposed for extracting bioactive compounds from aronia fruits and stems is in agreement with clean-label and sustainability principles intensively promoted in food and ingredient development.

Given the different behaviors of the analyzed waters depending on the nature of the plant material used (stems or fruits), additional studies are required in order to confirm the extraction properties of the respective natural mineral waters.

Overall, it can be concluded that natural mineral waters represent a promising alternative to conventional solvents, particularly for the valorization of plant by-products intended for food-related applications. The use of these waters contributes simultaneously to a greener extraction process and to the production of greener extracts.

## Figures and Tables

**Table 1:** Characteristics of commercial mineral waters used in extraction.

Natural Mineral Waters	pH (25 °C)	Dry Residue (180 °C) (mg/L)	Na^+^ (mg/L)	K^+^ (mg/L)	Ca^2+^ (mg/L)	Mg^2+^ (mg/L)	NO_3_^−^ (mg/L)	NO_2_^−^ (mg/L)	HCO_3_^−^ (mg/L)	CO_2_ (min.) (mg/L)
T *	5.59	1420	197.00	10.30	210.00	65.70	ND	ND	1260.00	2500
AQV **	min. 9.40	160	65.70	0.32	2.80	ND	<1	<0.05	158.00	-
AC **	7.89	132	1.34	ND	40.50	13.10	ND	ND	181.00	-
ACK **	8.10	222	0.70	ND	46.60	15.10	ND	0.90	232.30	-
PH **	7.30	371	7.10	1.62	97.70	10.10			324.00	-

ND = not declared; * medium mineral content; ** low mineral content. Natural mineral waters: T—Tușnad; AQV—Aquavia; AC—Aqua Carpatica; ACK—Aqua Carpatica Kids; PH—Perla Harghitei.

**Table 2:** Coding of the 42 samples.

Extraction Method	Solvent	Plant Material	
		Fruits	Stems
M	AD	F-AD-M	S-AD-M
	HA	F-HA-M	S-HA-M
	NMW	F-T-M, F-AQV-M, F-AC-M, F-ACK-M, F-PH-M	S-T-M, S-AQV-M, S-AC-M, S-ACK-M, S-PH-M
USRT	AD	F-AD-USRT	S-AD-USRT
	HA	F-HA-USRT	S-HA-USRT
	NMW	F-T-USRT, F-AQV-USRT, F-AC-USRT, F-ACK-USRT, F-PH-USRT	S-T-USRT, S-AQV-USRT, S-AC-USRT, S-ACK-USRT, S-PH-USRT
US50	AD	F-AD-US50	S-AD-US50
	HA	F-HA-US50	S-HA-US50
	NMW	F-T-US50, F-AQV-US50, F-AC-US50, F-ACK-US50, F-PH-US50	S-T-US50, S-AQV-US50, S-AC-US50, S-ACK-US50, S-PH-US50

**Table 3:** Pearson correlation coefficient matrix of four measured parameters for aronia (stems and fruits) extracts obtained using the five natural mineral waters, distilled water, and hydroalcoholic mixture.

		pH	EC	TDS	SAL
T	pH	1.0000	−0.0767	−0.1314	−0.1556
AQV		1.0000	0.3474	0.2290	0.3042
AC		1.0000	−0.6403	−0.6722	−0.7481
ACK		1.0000	−0.4532	−0.5431	−0.5622
PH		1.0000	−0.8869	−0.9014	−0.8888
AD		1.0000	−0.4545	−0.4233	−0.6443
HA		1.0000	−0.8671	−0.8587	−0.6820
T	EC		1.0000	0.9985	0.9968
AQV			1.0000	0.9924	0.9990
AC			1.0000	0.9991	0.9887
ACK			1.0000	0.9946	0.9920
PH			1.0000	0.9995	1.0000
AD			1.0000	0.9994	0.9741
HA			1.0000	0.9999	0.9557
T	TDS			1.0000	0.9997
AQV				1.0000	0.9970
AC				1.0000	0.9942
ACK				1.0000	0.9997
PH				1.0000	0.9996
AD				1.0000	0.9656
HA				1.0000	0.9605
T	SAL				1.0000
AQV					1.0000
AC					1.0000
ACK					1.0000
PH					1.0000
AD					1.0000
HA					1.0000

The color in the table shows the strength of association for Pearson correlation coefficient. (Red: Negative; Green: Positive).

**Table 4:** The main absorption bands from FTIR spectra of aronia extracts (S—stems; F—fruits).

Samples	Absorption Bands (cm^−1^)				References
7/S-M	3277–3314	1605–1642	1049–1052	598–647	[42,43,44,45,46,47]
7/S-US50	3277–3331	1631–1642	1049–1079	621–647	
7/F-M	3290–3340	1622–1634	1055–1066	613–647	
7/F-US50	3285–3315	1628–1634	1055–1075	613–640	
	stretching vibration of –OH groups (polyphenols, polysaccharides)	C=O asymmetric stretching vibration of extensively conjugated systems like flavonoids	C–O bonds of cyclic ethers and alcoholic groups from polyphenols	C–H stretching of aromatic groups	

S-M: stems—maceration; S-US50: stems—ultrasound-assisted extraction (50 °C); F-M: fruits—maceration; F-US50: fruits—ultrasound-assisted extraction (50 °C).

## Data Availability

The original contributions presented in this study are included in the article. Further inquiries can be directed to the corresponding authors.
